# Assessment of the Ferroptosis Regulators: Glutathione Peroxidase 4, Acyl-Coenzyme A Synthetase Long-Chain Family Member 4, and Transferrin Receptor 1 in Patient-Derived Endometriosis Tissue

**DOI:** 10.3390/biom14070876

**Published:** 2024-07-21

**Authors:** Lidia A. Mielke Cabello, Gabriela Meresman, Dogus Darici, Noelia Carnovale, Birthe Heitkötter, Miriam Schulte, Nancy A. Espinoza-Sánchez, Quang-Khoi Le, Ludwig Kiesel, Sebastian D. Schäfer, Martin Götte

**Affiliations:** 1 Department of Gynecology and Obstetrics, University Hospital of Muenster, 48149 Muenster, Germanyludwig.kiesel@ukmuenster.de (L.K.); seb.schaefer@alexianer.de (S.D.S.); 2 Institute of Biology and Experimental Medicine IBYME-CONICET, Buenos Aires C1428, Argentina; gabriela.meresman@gmail.com (G.M.);; 3 Institute of Anatomy and Molecular Neurobiology, University Hospital of Muenster, 48149 Muenster, Germany; 4 Gerhard-Domagk-Institute of Pathology, University Hospital of Muenster, 48149 Muenster, Germany

**Keywords:** endometriosis, ferroptosis, biomarkers, GPX4, ACSL4, TfR1, iron

## Abstract

Ferroptosis, an iron-dependent form of non-apoptotic cell death, plays a pivotal role in various diseases and is gaining considerable attention in the realm of endometriosis. Considering the classical pathomechanism theories, we hypothesized that ferroptosis, potentially driven by increased iron content at ectopic sites, may contribute to the progression of endometriosis. This retrospective case–control study provides a comprehensive immunohistochemical assessment of the expression and tissue distribution of established ferroptosis markers: GPX4, ACSL4, and TfR1 in endometriosis patients. The case group consisted of 38 women with laparoscopically and histologically confirmed endometriosis and the control group consisted of 18 women with other gynecological conditions. Our study revealed a significant downregulation of GPX4 in stromal cells of endometriosis patients (*M* = 59.7% ± 42.4 versus 90.0% ± 17.5 in the control group, t (54) = −2.90, *p* = 0.005). This finding aligned with slightly, but not significantly, higher iron levels detected in the blood of endometriosis patients, using hemoglobin as an indirect predictor (Hb 12.8 (12.2–13.5) g/dL versus 12.5 (12.2–13.4) g/dL in the control group; t (54) = −0.897, *p* = 0.374). Interestingly, there was no concurrent upregulation of TfR1 (*M* = 0.7 ± 1.2 versus 0.2 ± 0.4 for EM, t (54) = 2.552, *p =* 0.014), responsible for iron uptake into cells. Our empirical findings provide support for the involvement of ferroptosis in the context of endometriosis. However, variances in expression patterns within stromal and epithelial cellular subsets call for further in-depth investigations.

## 1. Introduction

Endometriosis (EM) is a prevalent, reproductive, estrogen-dependent, inflammatory disorder characterized by the presence of endometrial-like tissue growing abnormally outside the uterus [1]. Despite being first described almost a century ago, the precise causes, mechanisms, and pathways contributing to the development and progression of various EM subtypes remain unclear. With a global prevalence affecting around 10% of women, EM represents a significant health concern. Notably, 30 to 50% of symptomatic patients experience chronic pelvic pain and/or infertility, the two hallmark symptoms of EM [2,3]. 

EM can be categorized into three main subtypes based on its histopathology and anatomical locations: superficial endometriosis (SUP), deep infiltrating endometriosis (DIE), and ovarian endometriosis (OMA) [3,4]. Additionally, adenomyosis, found within the uterus, is characterized by endometrial tissue surrounded by smooth muscle cells within the myometrium. Interestingly, these various forms of EM often coexist, hinting at shared developmental pathways among the different types [3,5,6,7].

Various theories, including the widely accepted Sampson’s theory of retrograde menstruation, suggest that endometrial tissue fragments, cells, and protein-rich fluid can reflux through the fallopian tubes, ultimately reaching the peritoneal cavity, particularly within the pelvis [2,3,8]. These cells utilize a molecular strategy to adhere to the serosal surface and endure initial ischemic conditions. Consequently, the immune system perceives the misplaced endometrium as foreign, triggering an inflammatory response, aided by the high iron content in menstrual blood [3,6,9]. Individuals with a dysregulated inflammatory response may activate cancer-associated pathways like nuclear factor kappa-light-chain-enhancer of activated B cells (NF-κB), potentially due to genetic and epigenetic alterations [3]. This intriguingly mirrors the cancer-like characteristics exhibited by EM, despite its benign nature. Elevated levels of inflammatory mediators, hormones, and immune cells are observed in the tissue microenvironment and peritoneal fluid of patients with EM. These components contribute to survival, implantation, invasion, angiogenesis, and immunosurveillance evasion in endometriotic lesions [2,10].

The emergence and progression of DIE, which infiltrates deep organ tissue layers and structures, remain unclear. Although the notion that it is an extension of SUP is insufficient, the angiolymphatic dissemination and/or stem cell theory appear more fitting, despite its limitations [3,5,6]. The tissue injury and repair theory (TIAR) suggests that the elevated estrogen and progesterone resistance in adenomyosis triggers the oxytocin receptors, inducing hyperperistalsis and leading to changes in the endometrial–myometrial junctional barrier, myometrial injury, fibrosis, smooth muscle metaplasia, and microlesions, allowing cells to migrate like in cancer metastasis [5,7,11,12].

While bone-marrow-derived stem cells may play a role in rare cases, as in individuals with Mayer–Rokitansky–Küster–Hauser syndrome and in men, chronic inflammation and disease progression are more likely to occur due to immune responses and activation of certain pathways in an iron-rich environment [2,6,13].

Originally described as “oxytosis” in 1989, the term “ferroptosis”, coined by Dixon et al. in 2012, describes a distinct type of iron-dependent cell death. This emerging concept is gaining significance and attention for its implications in various diseases, including EM [9,14,15]. Ferroptosis is morphologically, biochemically, and genetically divergent from apoptosis, necrosis, and autophagy [16,17]. The influx of iron into cells via transferrin receptor 1 (TfR1) or through the divalent metal transporter 1 (DMT1) leads to an overproduction of reactive oxygen species (ROS) due to a redox imbalance, causing substantial lipid peroxidation in cell membranes and ultimately resulting in cell death [9,17].

Cellular iron is intricately regulated, with balance mechanisms controlling the stability and translation of specific iron-related messenger ribonucleic acids (mRNAs) coding for ferritin, transferrin, TfR1, DMT1, among others [18]. 

Physiologically, ingested iron is absorbed in duodenal enterocytes, a process regulated by hepcidin. Once in the bloodstream, iron can enter cells through TfR1, bound to transferrin in its ferric form (Fe^3+^), or through DMT1 in its divalent ferrous form (Fe^2+^) [18,19,20,21]. The metalloreductase six-transmembrane epithelial antigen of prostase 3 (STEAP3) converts the insoluble ferric form of iron into its soluble ferrous form, contributing to the cellular iron pool required for ferroptosis. Conversely, ferroportin (FPN) facilitates the efflux of iron from the cell [17,20,22]. In a state of inflammation, this delicate balance is disrupted.

Ferroptosis is tightly regulated at various levels, impacting both systemic and local iron homeostasis. This process can spread to adjacent cells rapidly, creating a propagating wave [15,17].

Several proteins and genes participate in inducing ferroptosis through diverse pathways. Identified antibodies, such as anti-TfR1, have proven effective in detecting cells undergoing ferroptosis in various contexts. A study by Feng et al. demonstrated the reliability of anti-TfR1 in indicating ferroptosis, applicable in both tissue sections and cell cultures [23].

Central to ferroptosis, glutathione peroxidase 4 (GPX4) and acyl-coenzyme A synthetase long-chain family member 4 (ACSL4) play pivotal roles as positive and negative regulators, respectively [24]. 

GPX4 inhibits the formation of lipid peroxides. It converts glutathione (GSH) into oxidized glutathione (GSSG) and reduces toxic lipid peroxides (L-OOH) to alcohols (L-OH). Inhibition or downregulation of GPX4 results in the accumulation of lipid peroxides and increases sensitivity to ferroptosis [9,15,19,25,26]. 

ACSL4 is crucial for fatty acid metabolism and lipid peroxidation [23,26]. Its role involves enhancing the content of polyunsaturated fatty acids (PUFAs) in phospholipids. Free PUFAs undergo esterification into membrane phospholipids and oxidation to transmit the ferroptosis signal. While ACSL4 is generally associated with ferroptosis, it may not be required in all cases, suggesting that ACSL4-depleted cells can undergo ferroptosis under specific circumstances. Inhibiting the expression of ACSL4 has been shown to reduce lipid peroxide accumulation and diminish ferroptosis [15,17,26]. 

Considering the classical pathomechanism theories of EM, we hypothesized that ferroptosis, potentially driven by increased iron content at ectopic sites, may contribute to the progression of the disease. We, therefore, studied the expression of established ferroptosis biomarkers—GPX4, ACSL4, and TfR1—by immunohistochemical analysis on EM tissue and on the eutopic endometrium of the controls.

## 2. Materials and Methods

### 2.1. Study Population

This retrospective case–control study included women aged 19 to 39. Inclusion criteria for the case group were laparoscopic and histological confirmation of EM in women who underwent surgery between 2021 and 2022 by the same surgeon at the Department of Gynecology and Obstetrics at the University Hospital in Muenster, Germany. Exclusion criteria were ages under 18 or over 50 years and concurrent diagnosis of malignancies. The severity of the disease was classified according to the rASRM score (Revised Classification of the American Society for Reproductive Medicine), ranging from stage I (minimal) to stage IV (severe). For a precise description, including the anatomical location, the size of the endometriotic lesions, and the involvement of the genital tract and adjacent organs, the ENZIAN and #ENZIAN classifications were utilized [27,28]. In the control group, we included women with other gynecological conditions such as abnormal uterine bleeding, polyps, or infertility who underwent endometrial scraping between 2017 and 2022. Patients with malignant disorders or histologically confirmed endometriosis were excluded from the control group. Anthropometric and clinical data were obtained from the patients’ records. The hemoglobin value was obtained from the standard pre-surgical blood analysis. 

### 2.2. Ethical Approval

The study was designed under consideration of the principles of the Helsinki Convention and was approved by the ethics committee of Westphalia-Lippe (1 IX Greb, from 19 September 2001, updated 2012).

### 2.3. Immunohistochemistry

The surgically obtained tissue samples were immediately fixed in 10% neutral-buffered formalin and embedded in paraffin according to the standard procedures. Serial sections of 5 µm were transferred to poly-l-lysine-coated slides and stained with hematoxylin and eosin for fine tissue examination. EM samples from different locations were selected for this study. Samples of eutopic endometrium from women without EM were used as controls. Dried slides were deparaffinized, rehydrated, and treated with target retrieval solution (pH 6.0) for 10 min in a steamer, followed by a wash in phosphate-buffered saline (PBS). Sections were blocked with peroxidase (Dako, Denmark A/S, Glostrup, Denmark) for 10 min, followed by a second block with 4% BSA (bovine serum albumin, Dako, Denmark A/S, Glostrup, Denmark) for 60 min. Sections were then incubated overnight at 4 °C with the following primary antibodies: mouse anti-TfR1 monoclonal antibody (1:20 = 0.05 mg/mL; RRID: AB_10966364; Cat# MA5-11441, ThermoFisher, Waltham, MA, USA), mouse anti-ACSL4 monoclonal antibody (1:100 = 0.029 mg/mL; RRID: AB_2787171; Cat# MA5-31543, ThermoFisher, Waltham, MA, USA), and rabbit anti-GPX4 recombinant monoclonal antibody (1:100 = 0.01 mg/mL; RRID: AB_2810103; Cat# MA5-32827, ThermoFisher, Waltham, MA, USA). Subsequently, sections were treated for 60 min with the corresponding secondary biotinylated antibody (goat anti-rabbit IgG, 1:200, B7389; Sigma-Aldrich, Burlington, MA, USA; or goat anti-mouse IgG, 1:200, BA-9200-1.5; Vector-Laboratories, Newark, CA, USA), followed by incubation with streptavidin–peroxidase (Roche Diagnostics GmbH, Mannheim, Germany) for 30 min. The binding was visualized by incubating sections with diaminobenzidine (DAB; Roche Diagnostics GmbH, Mannheim, Germany) and lightly counterstaining with hematoxylin before permanent mounting. A non-relevant antibody of the same species (mouse, rabbit) and the same immunoglobulin isotype (IgG1) was used as a negative control. We conducted positive controls with each antibody following the product information guidelines and referring to previously conducted studies: mouse testis tissue for GPX4, mouse epididymis for ACSL4, and mouse liver for TfR1 [19,23,29]. The tissue sections for the positive controls were sourced from the Institute of Biology and Experimental Medicine (IBYME-CONICET) in Buenos Aires, Argentina. 

### 2.4. Microscopic Evaluation 

A random subset of the slides (around 10%) was evaluated blindly by three independent investigators, two of whom are expert pathologists at the University Hospital in Muenster, Germany. The full dataset was evaluated by one pathologist.

Biopsy tissue sections were analyzed at ×40, ×100, and ×400 magnification using an Olympus BX51 Fluorescence microscope with an integrated imaging system from Hologic and photographed using a Sony E 18–55 mm f/3.5–5.6 OSS digital pathology scanner (Sony Corporation, Minato, Tokyo, Japan). 

For the immunohistochemical analysis, staining intensity of ACSL4, GPX4, and TfR1 was evaluated at ×400 magnification for each cell type. It was assessed according to a score of 0: light staining, 1: medium staining, and 2: intense staining.

Each of the used antibodies showed cytoplasmic staining of epithelial, stromal, or both cell types. For the quantitative analysis, a percentage ranging between 0% and 100% was assigned separately for each cell type.

### 2.5. Statistical Analysis 

Statistical analysis was conducted using SPSS v. 29.0 for Mac (SPSS, Chicago, IL, USA). The graphs were generated using GraphPad Prism v. 9 for Mac (GraphPad Software, Boston, MA, USA). For descriptive analysis, mean and standard deviations were calculated for normally distributed continuous variables, while median and quartiles were used for non-normally distributed variables. Additionally, categorical variables were presented with absolute and relative frequencies.

Two-sided t-tests for independent samples with Bonferroni correction were applied to compare the protein expression between groups. Additionally, non-parametric Mann–Whitney U-tests were conducted for non-normally distributed outcomes. Pearson’s correlation was used to analyze the correlation between the variables. The clinical characteristics were analyzed as categorical variables by the Chi-squared test. All statistical analyses were performed with a significance value of alpha = 0.05, where a *p*-value < 0.05 was considered statistically significant. A power analysis was conducted with an alpha = 0.05, power = 0.80, and effect size d = 0.80.

## 3. Results

### 3.1. Patient Characteristics

The anthropometric and clinical characteristics of the women included in this study are presented in Table 1 and Table 2. Out of a total of 56 women, 38 had laparoscopically and histologically confirmed EM. The collected samples were derived from different anatomical locations: pelvic sidewall (9), sacrouterine ligament (5), bladder peritoneum (5), rectovaginal space (5), rectum (3), endometrioma (3), vaginal wall (2), pararectal space (2), diaphragm (1), abdominal wall (1), appendix vermiformis (1), and ureter (1). The samples were analyzed altogether (i.e., Table 3). The interrater reliability (one-way random model) between all three raters was CCC = 0.942, 95% CI: 0.916; 0.960, *p* < 0.001, showing high reliability according to Landis and Koch [30]. In the control group of 18 women, the absence of EM was histologically confirmed. No significant correlations between the location and stage of the disease were found. 

We observed slightly, but not significantly higher, hemoglobin levels in patients with EM, serving as an indirect predictor of iron levels (Hb 12.8 (12.2–13.5) g/dL versus 12.5 (12.2–13.4) g/dL in the control group; t (54) = −0.897, *p* = 0.374). The complete clinical data are available in the Supplementary Materials (i.e., Supplementary Table S1).

The power analysis (alpha = 0.05, power = 0.80, and effect size d = 0.80) indicated a minimum sample size of 26 per group. Therefore, the current study was slightly underpowered.

### 3.2. Staining Pattern of ACSL4, GPX4, and TfR1

**Figure 1:**
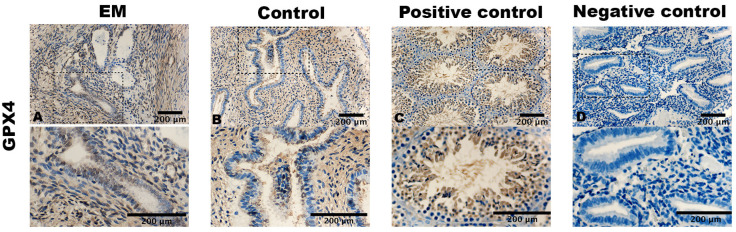
Expression of GPX4. (**A**) Ectopic endometrium (vaginal wall) of endometriosis patient. (**B**) Eutopic endometrium of control. (**C**) Mouse testis as positive control. (**D**) Eutopic endometrium of control treated with an unrelated monoclonal antibody of the same isotype as negative control. ×400 magnification. Scale bar = 200 μm.

ACSL4, GPX4, and TfR1 exhibit cytoplasmic staining in cells. GPX4 displayed the strongest expression in terms of both the percentage and staining intensity of cells (i.e., Figure 1). Staining was prominently observed in the cytoplasm of stromal cells and in the epithelial cells of the glands. In the case of the endometriotic lesion samples, staining was observed throughout the gland, even extending to some nuclei (i.e., Figure 1A).

However, in the control samples, staining was predominantly restricted to the luminal epithelium of the glands (i.e., Figure 1B).

**Figure 2:**
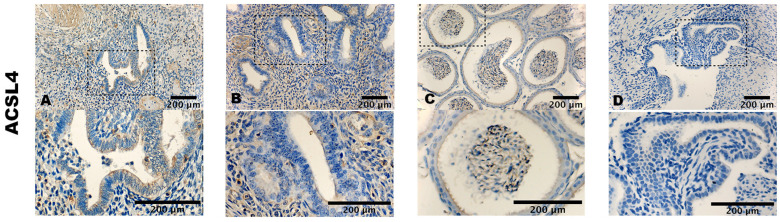
Expression of ACSL4. (**A**) Ectopic endometrium (ureter) of endometriosis patient. (**B**) Eutopic endometrium of control. (**C**) Mouse epididymis as positive control. (**D**) Ectopic endometrium (sacrouterine ligament) treated with an unrelated monoclonal antibody of the same isotype as negative control. ×400 magnification. Scale bar = 200 μm.

**Figure 3:**
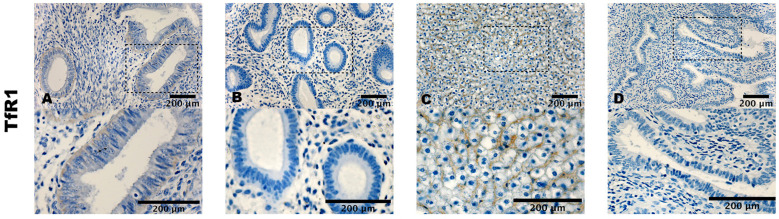
Expression of TfR1. (**A**) Ectopic endometrium (abdominal wall) of endometriosis patient. (**B**) Eutopic endometrium of control. (**C**) Mouse liver as positive control. (**D**) Eutopic endometrium of control treated with an unrelated monoclonal antibody of the same isotype as negative control. ×400 magnification. Scale bar = 200 μm.

ACSL4 exhibited a similar staining pattern in terms of both cell count and intensity (i.e., Figure 2A,B). In the case of TfR1, very mild to almost imperceptible staining was observed in the cytoplasm of both stromal and epithelial cells (i.e., Figure 3A,B).

The positive controls displayed a cytoplasmatic staining of the cells of mouse testis (i.e., Figure 1C), mouse epididymis (i.e., Figure 2C), and mouse liver (i.e., Figure 3C). Negative controls showed no staining (i.e., Figure 1D, Figure 2D and Figure 3D). 

### 3.3. GPX4, ACSL4, and TfR1 Expression Levels

**Figure 4:**
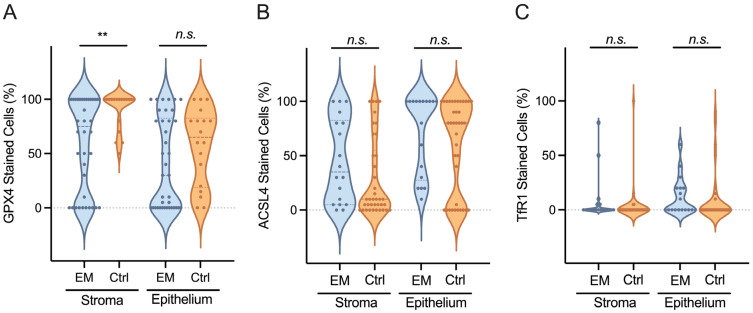
Violin plots of the percentage (%) of stained cells. (**A**) Expression of GPX4 in stromal and epithelial cells of patients with endometriosis (EM) and controls (Ctrl). (**B**) Expression of ACSL4 in stromal and epithelial cells of endometriosis patients and controls. (**C**) Expression of TfR1 in stromal and epithelial cells of endometriosis patients and controls. ** *p* < 0.01; n.s. = not statistically significant.

The strongest expression was observed in stromal cells with the GPX4 antibody. The percentage of stained stromal cells was higher in the control group with M = 90.0% ± 17.5 versus 59.7% ± 42.4 in the EM group, t (54) = −2.90, *p* = 0.005 (i.e., Figure 4, Supplementary Table S2). The intensity of the staining of stromal cells was stronger in the EM group with M = 1.0 ± 0.7 versus 1.4 ± 0.6 in the control group, t (54) = 2.22, *p* = 0.031 (i.e., Table 4).

Comparing the expression of the different antibodies between EM patients and the control group, we observed a similar staining for GPX4 and ASCL4. Both antibodies denote a strong staining intensity of stromal cells M = 1.0 ± 0.7 for EM and 1.4 ± 0.6 for controls, t (54) = −2.22, *p* = 0.031 with GPX4 and M = 1.1 ± 0.8 for EM and 1.2 ± 0.4 for controls, t (54) = −0.809, *p* = 0.422 with ACSL4. In the case of epithelial cells, M = 1.2 ± 1.1 for EM and 1.3 ± 0.7 for controls, t (54) = −1.235, *p* = 0.815 with GPX4 and M = 1.1 ± 0.9 for EM and 1.1 ± 0.6 for controls, t (54) = 0.085, *p* = 0.932 with ACSL4.

TfR1 showed a very low expression level. The staining intensity was stronger in the stromal cells of the control group with M = 0.7 ± 1.2 versus 0.2 ± 0.4 for EM, t (54) = 2.552, *p* = 0.014 (i.e., Table 4).

We further analyzed the association between the different antibodies using correlation-based analyses. In all patients, the strongest correlations were found between GPX4 and ACSL4, where r = 0.758, *p* < 0.001 for the percentage of stained stromal cells and r = 0.672, *p* < 0.001 for the intensity of the staining of stromal cells, and r = 0.694, *p* < 0.001 for the percentage of stained epithelial cells and r = 0.714, *p* < 0.001 for the intensity of the staining of epithelial cells.

## 4. Discussion

The pathomechanism of EM remains unclear despite years of intensive research. Various theories have been proposed to explain its origin and development; however, numerous uncertainties persist. 

Considering the classical pathomechanism theories of EM, we hypothesized that ferroptosis, potentially driven by increased iron content at ectopic sites, may contribute to the progression of the disease. Our findings showed a significant downregulation of GPX4 in the stromal cells of EM patients, linked to a slightly higher hemoglobin value as an indirect predictor of the iron level, as the amount of iron in hemoglobin accounts for about two-thirds of the mass of iron in the human body [18].

GPX4 and hemoglobin play crucial roles in managing oxidative stress. Hemoglobin can generate reactive oxygen species (ROS), which trigger ferroptosis, while GPX4 helps mitigate the damage caused by ROS, particularly lipid peroxides that can damage cell membranes and lead to cell death. In the context of EM, higher levels of hemoglobin and iron may trigger ferroptosis, potentiated by a downregulation of GPX4 [31].

Both systemic and local iron homeostasis influence cell sensitivity to ferroptosis, which can propagate rapidly to adjacent cells [15,17,32]. Ferroptosis operates through two major pathways: the extrinsic or transporter-dependent pathway and the intrinsic or enzyme-regulated pathway [16,17,26]. As mentioned above, cellular iron is intricately regulated; this delicate balance is disrupted in a state of inflammation [18].

Previous research has demonstrated iron accumulation in both endometriotic lesions and the peritoneal fluid of patients with EM [2,33,34,35]. Consistent with other studies, which either detected higher iron levels in endometriosis patients or found no significant difference, our findings suggest that patients with EM exhibit slightly, but not significantly, higher systemic iron levels compared to the control group [13,36,37]. This finding can be attributed to the fact that most patients with EM are on hormonal therapy that inhibits menstruation, potentially explaining the slightly higher hemoglobin levels. As the day of the cycle was not documented, it presents a potential source of bias, given that hemoglobin levels are known to decrease in women during menstruation. 

Paradoxically, our results showed a downregulation of TfR1 in stromal cells of EM patients compared to the controls. This outcome could be attributed to the previously identified aberrant iron transport discussed in several other studies [2,37]. The significant presence of iron within endometriotic lesions may trigger a downregulation of TfR1 as a protective mechanism for cells against excessive iron influx. However, it is crucial to acknowledge that TfR1 is not the sole player in iron transport. Other studies conducted in patients with EM have shown that iron overload induces the increased expression of two subtypes of DMT1, which is also responsible for iron influx into cells [38,39]. Akashi et al. found a downregulation in TfR1 and FPN and an upregulation of DMT1 in samples of ovarian endometriosis (OMA) and clear cell carcinoma [40].

The influx of high levels of iron into the cells induces a lethal accumulation of ROS through the Fenton reaction, resulting in a redox imbalance between oxidants and antioxidants and leading to the extensive lipid peroxidation of cell membranes and, ultimately, cell death [9,17,20,21,26]. 

GPX4 inhibits the formation of lipid peroxides. Therefore, the inhibition or downregulation of GPX4 results in the accumulation of lipid peroxides and increases sensitivity to ferroptosis [9,15,19,25,26]. Our study identified a significant downregulation of GPX4 in stromal cells of EM patients, suggesting a potential link between ferroptosis and the pathomechanism of EM. Recent findings also propose the involvement of different genetic variants of GPX4 in the pathomechanism of EM [41,42]. The observation within stromal cells adds a layer of complexity, highlighting the distinct involvement of both cell types: epithelial and stromal, and deepening the intricacy of understanding the disease. 

Queckbörner et al., utilizing single-cell RNA sequencing, identified ten distinct stromal cell subpopulations, establishing cell cluster diversity and highlighting the complexity of the endometrial stromal compartment [43,44]. Zhang et al. also described endometrial stromal cell subpopulations and observed that iron overload in ectopic endometrial stromal tissue, followed by ferroptosis, promotes fibrosis and adhesion [45]. Furthermore, Akashi et al. described a highly proliferative endometrial epithelium infiltrating the stroma with elevated Ki-67 expression in patients with EM, emphasizing the proliferative nature of the disorder [40].

In the current comprehension of EM pathophysiology, these observations indicate that distinct subpopulations of stromal cells react to ferroptosis triggered by elevated iron levels. Consequently, a microenvironment characterized by inflammation, hypoxia, and angiogenesis emerges, fostering the proliferation and infiltration of epithelial cells. This dynamic contributes to the formation and expansion of endometriotic lesions. The intricate processes at play, mediated through diverse inflammatory pathways, that require further and deeper investigation, may induce ferroptosis-triggered fibrosis, typical of deep infiltrating lesions.

Supporting our hypothesis, Alvarado-Díaz et al. demonstrated that exposing isolated endometrial stromal cells to iron excess stimulates the pro-inflammatory NF-κB pathway, enhancing the migration ability of endometriotic cells by promoting the expression of matrix metalloproteinases (MMPs) and exacerbating inflammation, angiogenesis, and cell adhesion [3,46,47]. Li et al. demonstrated that inducing ferroptosis in endometriotic stromal cells increases the expression of pro-inflammatory and angiogenic cytokines, such as interleukin 8 (IL-8) and vascular endothelial growth factor A (VEGFA) [10]. Other studies have highlighted the contribution of elevated levels of inflammatory mediators, hormones, and immune cells as cyclooxygenase-2, interleukin-β, interleukin-6, IL-8, tumor necrosis factor alpha, prostaglandin E2, and estradiol in the tissue microenvironment and the peritoneal fluid of patients with EM in the survival, implantation, invasion, growth, angiogenesis, immunosurveillance evasion, and establishment of endometriotic lesions [10,45,48,49,50,51]. Furthermore, Akashi et al. observed M2 macrophages engulfed with iron in the stroma of OMA, linking excessive erythrophagocytosis, iron overload, and ferroptosis in macrophages to the chronic inflammatory mechanism of EM [40,52,53].

Regarding ACSL4, which is generally associated with promoting ferroptosis, our study revealed a strong correlation between ACSL4 and GPX4 in both the stromal and epithelial cells of patients with EM and in the controls. This finding highlights the delicate equilibrium between induction and inhibition, as ACSL4 and GPX4 are well established as positive and negative regulators of ferroptosis, respectively [24]. 

As a limitation of our pilot study, we analyzed a small group of patients, comparing the eutopic endometrium of non-endometriosis control women with ectopic lesions of endometriosis patients. While we consider this an appropriate control for comparing healthy with diseased tissue, the inclusion of eutopic endometrium from endometriosis patients could have provided further insights into potential pathogenetic mechanisms. Moreover, endometriosis is a hormone-dependent disease characterized by chronic inflammation [1,2]. Although subgroup analysis according to hormonal therapy or analgesics did not reveal statistically significant differences in the expression of ferroptosis markers, the use of these drugs could have acted as a possible confounder. Larger sample sizes are needed to validate these results. Additionally, the hormonal status of the patients was unknown, as it is not routinely tested. 

While we identified GPX4 as dysregulated in EM, alternative pathways of ferroptosis surveillance independent of GPX4 have been described in oncological settings. Among these, the phospholipid-modifying enzymes MBOAT1 and MBOAT2 suppress ferroptosis [54] and are expressed in an estrogen- and androgen-dependent manner, marking them as worthwhile candidates for future investigations of the steroid-related regulation of ferroptosis in EM. 

Our observation of decreased GPX4 expression in endometriotic tissue compared to control tissue suggests a possible involvement of ferroptosis in EM. While this contrasts with previous suggestions of potential ferroptosis resistance in the disease [13], it also opens up the possibility of the pharmacological inhibition of ferroptosis in EM. Indeed, inhibitors of ferroptosis such as Baicalin, Selenium, Dexmedetomidine, Dexpramipexole, and several natural compounds have been proposed as emerging treatments targeting key regulators of ferroptosis in the context of neurodegenerative diseases and strokes [55,56,57] and may be worth evaluating in preclinical models of EM. 

## 5. Conclusions

In summary, our investigation into the progression of EM reveals a complex interplay involving ferroptosis, disrupted iron metabolism, and intricate inflammatory responses. The downregulation of GPX4 in stromal cells, along with elevated systemic iron levels and the complex regulation of iron transporters in patients with EM, suggests a potential link between ferroptosis and the disease’s pathomechanism. 

The influx of high iron levels triggers ferroptosis, leading to a cascade of events, including inflammation, hypoxia, angiogenesis, and eventual cell death. This microenvironment supports the proliferation and infiltration of epithelial cells, contributing to the establishment and growth of endometriotic lesions. Paradoxically, TfR1 downregulation, potentially as a protective mechanism, adds another layer of complexity to iron regulation in EM.

Understanding the involvement of ferroptosis and iron dysregulation in EM opens up avenues for further research and potential therapeutic interventions targeting these pathways. It underscores the necessity for a comprehensive exploration of the molecular mechanisms of EM, underlying iron transport, ferroptosis, cell differentiation, and genetic variants of implicated proteins to unravel the fairly unknown pathophysiology of EM.

## Figures and Tables

**Table 1:** Anthropometric and clinical characteristics of cases and controls.

	All Women N = 56 Median (25th–75th Percentile)	Endometriosis n = 38 Median (25th–75th Percentile)	Controls n = 18 Median (25th–75th Percentile)	*p*-Value
Age	30.5 (26.0–35.0)	29.8 (25.0–37.0)	32.1 (30.5–36.3)	n.s.
BMI (kg/m^2^)	24.9 (21.0–26.8)	24.7 (21.0–31.9)	25.4 (21.0–29.5)	n.s.
Hb (g/dL)	12.7 (12.2–13.5)	12.8 (12.2–13.5)	12.5 (12.2–13.4)	n.s.

BMI = body mass index; Hb = hemoglobin; *p* < 0.05 was considered statistically significant. n.s. = not statistically significant.

**Table 2:** Clinical characteristics of cases and controls.

	All Women N = 56	Endometriosis n = 38	Controls n = 18	*p*-Value
Past hormone therapy	39	30	9	0.028
Current hormone therapy	17	16	1	0.005
Dysmenorrhea	35	35	0	-
Infertility	17	5	12	0.000
Irregular cycle	22	20	2	0.003
Bleeding disorder	25	16	9	n.s.
Analgesics	26	26	0	-

Past hormone therapy = gestagene pill, combined oral contraceptive pill or hormonal intrauterine device >3 months; current hormone therapy = gestagene pill, combined oral contraceptive pill or hormonal intrauterine device; infertility = failure to achieve a pregnancy after 12 months or more of regular unprotected sexual intercourse (WHO, 2023); irregular cycle = shortest to longest cycle variation > 10 days; bleeding disorder = menorrhagia, metrorrhagia, menometrorrhagia, polymenorrhea, hypermenorrhea, oligomenorrhea, and intermenstrual bleeding; analgesics: NSAR (non-steroidal anti-inflammatory drug), opioids. *p* < 0.05 was considered statistically significant. n.s. = not statistically significant.

**Table 3:** Endometriosis stage and classification.

	rASRM I n = 10	rASRM II n = 9	rASRM III n = 9	rASRM IV n = 10
SUP	10	9	8	9
DIE	2	5	9	10
P	8	9	9	10
O	0	3	6	5
T	0	2	5	8
A	1	1	5	9
B	0	4	7	10
C	1	1	4	9
FA	9	8	9	10
FB	0	1	2	1
FI	0	0	1	2
FU	0	0	0	1
F	1	1	1	0

SUP = superficial endometriosis; DIE = deep infiltrating endometriosis; P = peritoneum; O = ovary; T = tube; A = rectovaginal space, vagina, retrocervical Area; B = acrouterine ligaments, cardinal ligaments, pelvic sidewall; C = rectum; FA = adenomyosis; FB = blader; FI = intestinum; FU = ureter; F = diaphragm, lung, nerve. The ENZIAN classification describes the deep infiltrating endometriosis lesions with A, B, C, FA, and F. The #ENZIAN classification is an extension of the ENZIAN classification, that also describes superficial lesions with P, O, T, A, B, C, FA, FB, FI, FU, F (…).

**Table 4:** Staining intensity of GPX4, ACSL4, and TfR1.

	Endometriosis n = 38	Controls n = 18	*p*-Value
GPX4 stromal cells	1.0 ± 0.7	1.4 ± 0.6	0.031
GPX4 epithelial cells	1.2 ± 1.1	1.3 ± 0.7	n.s
ACSL4 stromal cells	1.1 ± 0.8	1.2 ± 0.4	n.s
ACSL4 epithelial cells	1.1 ± 0.9	1.1 ± 0.6	n.s
TfR1 stromal cells	0.2 ± 0.4	0.7 ± 1.2	0.014
TfR1 epithelial cells	0.1 ± 0.3	0.3 ± 0.5	n.s

0 = light staining; 1 = medium staining; 2 = intense staining; *p* < 0.05 was considered statistically significant. n.s. = not statistically significant.

## Data Availability

All data generated for the manuscript are included in the study and are available upon request.

## Supplementary Materials

The following supporting information can be downloaded at: https://www.mdpi.com/article/10.3390/biom14070876/s1. Supplementary Table S1—Data; Supplementary Table S2—Expression levels of GPX4, ACSL4, and TfR1 in (%).
