# Supplementary material for: Assessment of the Ferroptosis Regulators: Glutathione Peroxidase 4, Acyl-Coenzyme A Synthetase Long-Chain Family Member 4, and Transferrin Receptor 1 in Patient-Derived Endometriosis Tissue
Source: Biomolecules. 2024 Jul 21;14(7):876. doi: 10.3390/biom14070876 (PMC11274870; doi:10.3390/biom14070876)
Supplement: Supplementary file 1 [file biomolecules-14-00876-s001.zip › biomolecules-3034569-supplementary Table S1.pdf]

CASES			
Anatomical localisation	Age	Surgery year	Previous EM surgerie
Diaphragm	21	2022	1
Rectum	33	2022	1
Sacrouterine ligament	23	2022	0
Bladder peritoneum	35	2022	0
Rectovaginal space	23	2022	0
Sacrouterine ligament	28	2022	2
Pelvic sidewall	19	2022	0
Pelvic sidewall	29	2022	0
Pelvic sidewall	24	2022	0
Bladder peritoneum	34	2022	1
Rectovaginal space	28	2022	1
Rectovaginal space	33	2022	0
Pelvic sidewall	23	2021	1
Abdominal wall	33	2022	1
Pelvic sidewall, sacrouterine ligamen	25	2022	9
Pelvic sidewall	34	2022	6
Sacrouterine ligament	26	2021	0
Rectovaginal space	36	2022	0
Pelvic sidewall	25	2021	0
Endometrioma	32	2022	1
Appendix	33	2022	1
Endometrioma	34	2022	1
Endometrioma	33	2022	0
Bladder peritoneum	37	2022	0
Ureter	27	2022	3
Pelvic sidewall	37	2022	1
Bladder peritoneum	30	2022	1
Bladder peritoneum	37	2022	3
Sacrouterine ligament	31	2022	0
Vaginal wall	31	2022	1
Pararectal space	20	2022	0
Pararectal space	32	2022	0
Pelvic sidewall	25	2022	0
Pelvic sidewall	31	2022	1
Rectum	38	2022	5
Vaginal wall	39	2021	0
Rectum	26	2021	1
Rectovaginal space	26	2021	1
CONTROLS			
Endometrium	36	2018	0
Endometrium	31	2018	0
Endometrium	29	2017	0
Endometrium	35	2017	0
Endometrium	36	2018	0
Endometrium	32	2022	0
Endometrium	26	2022	0
Endometrium	19	2022	0
Endometrium	36	2022	0

Endometrium	31	2022	0
Endometrium	31	2020	0
Endometrium	32	2021	0
Endometrium	32	2021	0
Endometrium	37	2021	0
Endometrium	23	2021	0
Endometrium	37	2021	0
Endometrium	38	2021	0
Endometrium	37	2022	0

**Past hormone therap**	**Actual hormone therap**	**Dysmenorrhe**	**Dyspareunia**	**Dysuria**	**Dyschezia**
1	1	1	0	1	1
0	0	1	0	0	0
1	0	1	1	0	1
1	1	1	1	0	0
1	1	1	1	0	1
1	0	1	1	0	0
1	1	1	1	0	0
1	0	1	1	0	0
1	0	1	1	1	0
1	1	1	1	1	1
1	1	1	1	0	1
0	0	1	1	0	1
1	1	1	1	0	0
1	0	1	1	0	1
1	1	0	0	0	0
1	0	1	0	1	1
0	0	1	0	0	0
1	0	1	1	0	0
1	1	1	0	0	1
0	0	1	0	0	0
1	0	1	1	0	1
1	0	1	0	0	0
1	1	1	0	0	0
1	1	1	0	0	0
0	1	1	0	1	0
0	0	0	0	0	0
1	0	1	0	1	1
1	1	0	0	0	0
1	0	1	1	0	1
1	1	1	1	1	0
0	1	1	0	1	1
1	0	1	0	0	1
1	0	1	1	0	0
1	0	1	0	0	0
1	0	1	1	0	1
1	1	1	1	0	1
1	0	1	0	1	0
0	0	1	1	1	1
0	0	0	0	0	0
1	0	0	0	0	0
1	0	0	0	0	0
0	0	0	0	0	0
1	0	0	0	0	0
0	0	0	0	0	0
0	0	0	0	0	0
1	0	0	0	0	0
1	0	0	0	0	0

1	0	0	0	0	0
0	1	0	0	0	0
1	0	0	0	0	0
1	0	0	0	0	0
1	0	0	0	0	0
0	0	0	0	0	0
0	0	0	0	0	0
0	0	0	0	0	0
0	0	0	0	0	0

Haematochezia	Haematuria	Endo-Belly	Sterility	Pregnancies	Irregular cycle	Regular cycle
0	0	0	0	G0	0	1
0	0	0	0	G2 P2	0	1
0	0	0	0	G0	1	0
0	0	0	0	G0	0	1
0	0	1	0	G0	1	0
0	0	0	0	G1 P1	1	0
0	0	0	0	G0	1	0
0	0	0	0	G0	1	0
0	0	0	0	G1 P0 A1	0	1
0	0	1	0	G0	1	0
0	0	0	0	G0	1	0
0	0	1	0	G1 P1	1	0
0	0	1	0	G0	0	1
0	0	1	0	G2 P2	1	0
0	0	0	0	G0	0	0
0	0	0	0	G2 P2	0	1
0	0	0	0	G0	0	1
0	0	0	0	G0	0	1
0	0	0	0	G0	1	0
1	1	1	0	G0	1	0
0	0	1	1	G0	0	1
0	0	1	0	G0	1	0
0	0	0	0	G0	1	0
0	0	0	0	G0	1	0
0	0	0	0	G0	1	0
0	0	0	1	G2 P1 A1	0	1
0	1	0	1	G0	0	1
0	0	0	0	G0	0	0
1	0	0	0	G0	1	0
0	0	0	0	G0	0	1
1	0	1	0	G0	1	0
0	0	0	1	G0	0	1
0	0	0	0	G0	1	0
0	0	0	1	G0	0	1
0	0	1	0	Go	1	0
1	0	1	0	G0	1	0
0	1	0	0	G0	0	1
0	0	0	0	G0	0	0
0	0	0	1	G1 P1	0	1
0	0	0	1	G0	0	1
0	0	0	1	G0	0	1
0	0	0	1	G0	0	1
0	0	0	1	G0	0	1
0	0	0	1	G0	0	1
0	0	0	1	G0	0	1
0	0	0	0	G0	0	1
0	0	0	1	G0	0	1

0	0	0	1	G0	1	0
0	0	0	0	G4 P2 A2	0	1
0	0	0	1	G0	0	1
0	0	0	0	G2 P2	0	1
0	0	0	0	G5 P3 A2	0	1
0	0	0	0	G0	0	1
0	0	0	0	G1 P1	0	1
0	0	0	1	G0	0	1
0	0	0	1	G1 P1	1	0

**Bleeding disorde**	**Back pain**	**Obstipatio**	**Diarrhea**	**Mood swing**	**Other complaint**	**Analgetics**
1	0	1	0	1	1	1
1	0	0	0	1	1	1
0	1	0	1	0	1	1
0	0	0	0	0	1	1
1	0	0	1	1	0	1
0	1	0	0	0	1	0
0	0	0	0	1	1	1
0	0	0	1	0	1	1
0	0	1	0	1	0	1
0	0	0	1	1	1	1
1	0	0	0	1	0	1
1	0	0	1	0	1	1
0	0	0	0	0	1	0
0	0	0	0	1	1	1
0	0	0	0	0	1	1
1	0	1	1	0	0	0
0	0	0	1	0	1	1
1	0	0	0	0	0	1
1	0	0	1	0	1	1
0	0	1	0	1	1	1
0	0	1	0	1	1	1
1	0	0	0	0	1	0
1	0	0	0	0	0	1
1	0	0	1	0	0	0
1	0	1	0	0	1	1
0	0	0	0	0	0	0
0	0	0	0	0	0	0
0	0	0	0	0	1	0
1	1	0	0	1	1	1
0	1	0	0	0	1	1
1	0	1	1	1	1	1
0	0	0	0	0	1	1
0	0	0	0	0	1	0
0	0	0	0	0	0	0
1	1	1	0	1	1	1
1	0	1	0	1	1	1
0	0	1	0	0	1	0
0	0	0	0	0	1	0
1	0	0	0	0	0	0
1	0	0	0	0	0	0
0	0	0	0	0	0	0
0	0	0	0	0	0	0
0	0	0	0	0	0	0
0	0	0	0	0	0	0
1	0	0	0	0	0	0
1	0	0	0	0	0	0
0	0	0	0	0	0	0

0	0	0	0	0	0	0
1	0	0	0	0	0	0
0	0	0	0	0	0	0
1	0	0	0	0	0	0
1	0	0	0	0	0	0
1	0	0	0	0	0	0
0	0	0	0	0	0	0
0	0	0	0	0	0	0
1	0	0	0	0	0	0

Smoking	Family medical history of E	Height	Weight	BMI	Hb-value (g/dL
1	1	165	65	24	14
0	0	168	63	22	12,2
0	0	170	55	19	13,5
0	0	185	72	21	13,8
0	0	174	74	24	14,7
0	0	165	60	22	12,9
0	0	172	79	27	12,8
0	0	172	75	25	12,6
0	0	163	63	24	12,4
0	1	171	76	26	13,5
1	0	168	56	20	11,4
0	0	158	60	24	12,7
0	1	173	60	20	11,4
0	0	165	70	26	12,2
0	0	170	115	40	13,8
0	0	162	82	31	12,9
0	0	150	47	21	11,9
0	0	168	81	29	14,2
0	0	165	56	21	13
0	0	168	78	28	12
0	0	169	58	20	12,9
0	0	172	72	24	11,5
0	0	167	57	20	13,3
0	0	174	69	23	12,7
0	0	160	76	30	12,5
1	0	174	70	23	12,9
0	0	168	115	41	14,6
0	0	173	68	23	13,2
0	0	170	68	24	14,4
0	0	172	58	20	11,6
0	0	168	53	19	11,8
0	0	166	59	21	13,6
0	1	166	65	24	9,4
0	0	180	65	20	13,6
0	0	167	63	23	13,2
0	0	160	106	41	11,7
0	0	171	60	21	13,5
0	0	160	67	26	12,9
1	0	170	85	29	12,7
0	0	173	108	36	13,2
0	0	180	75	23	12,4
0	0	167	64	23	13,2
0	0	176	73	22	13,5
1	0	164	59	22	12,3
0	0	175	57	19	6,9
0	0	174	64	21	11,8
0	0	176	68	22	13,3

0	0	181	102	31	13,2
0	0	160	63	25	13,7
0	0	172	95	32	13,2
0	0	167	53	19	13,1
0	0	174	89	29	13,7
0	0	175	130	42	11
0	0	170	54	19	12,6
0	0	172	63	21	13,6
0	0	173	68	23	11,6

**Lower abdominal pain**
0
0
0
0
0
0
0
0
0
0
0
0
0
0
0
0
0
0
0
0
0
0
0
0
0
0
0
0
0
0
0
0
0
0
0
0
0
0
0
1
1
1
0
1
0
0
1

	1
	1
	1
	1
	0
	0
	1
	0
	0
