# Supplementary material for: Assessment of the Ferroptosis Regulators: Glutathione Peroxidase 4, Acyl-Coenzyme A Synthetase Long-Chain Family Member 4, and Transferrin Receptor 1 in Patient-Derived Endometriosis Tissue
Source: Biomolecules. 2024 Jul 21;14(7):876. doi: 10.3390/biom14070876 (PMC11274870; doi:10.3390/biom14070876)
Supplement: Supplementary file 1 [file biomolecules-14-00876-s001.zip › biomolecules-3034569-supplementary Table S2.pdf]

Supplementary Table 2 - Expression levels of GPX4, ACSL4, and TfR1 in (%).

	**Endometriosis n = 38**	**Controls n = 18**	***p*-value**
GPX4 stromal cells	60.0 ± 42.4	90.0 ± 17.5	0.005
GPX4 epithelial cells	40.8 ± 42.0	58.0 ± 35.2	n.s.
ACSL4 stromal cells	60.7 ± 41.7	71.1 ± 36.6	n.s.
ACSL4 epithelial cells	28.7 ± 36.1	45.3 ± 38.6	n.s.
TfR1 stromal cells	8.6 ± 22.4	11.9 ± 17.2	n.s.
TfR1 epithelial cells	3.3 ± 16.3	8.3 ± 21.4	n.s.

$p < 0.05$ was considered statistically significant.
